# Redefining safety standards: a large-scale comparative analysis of bovine versus murine models for medical device embryotoxicity testing

**DOI:** 10.1007/s10815-026-03891-5

**Published:** 2026-05-02

**Authors:** Raquel Romar, Jon Romero-Aguirregomezcorta, Maria Maroto, Alfonso Gutiérrez-Adán, Pilar Coy

**Affiliations:** 1 Department of Physiology, International Excellence Campus for Higher Education and Research (Campus Mare Nostrum), University of Murcia, 30100 Murcia, Spain; 2 Institute for Biomedical Research of Murcia, IMIB Pascual Parrilla, Murcia, Spain; 3 Embryocloud SL, Murcia, Spain; 4 Department of Reproduction, Instituto Nacional de Investigación y Tecnología Agraria y Alimentaria, Madrid, Spain

**Keywords:** Embryotoxicity, Human culture media, Assisted reproduction, Mouse embryo assay, Bovine embryo assay

## Abstract

**Purpose:**

To overcome the limited sensitivity of the standard mouse embryo assay (MEA) for embryotoxicity screening of assisted reproduction devices and to assess the bovine embryo assay (BEA) as a suitable alternative.

**Methods:**

In a large comparative laboratory study, sperm selection, fertilization, and embryo culture media from two suppliers (Vitrolife and Genea Biomedx, respectively) were used to generate bovine blastocysts from cumulus–oocyte complexes obtained from slaughterhouse ovaries and inseminated with frozen semen from the same bull (BEA). Bovine culture media were used for the control group. BEA assessed the following in the three groups: cleavage; blastocyst development and kinetics; post-warming re-expansion/hatching; total cell number; inner cell mass (ICM) and trophectoderm (TE) allocation; and ICM/TE ratio. In parallel, the culture media from both suppliers were tested using mouse cumulus-oocyte complexes that had been inseminated with epididymal sperm (IVF-MEA) and in vivo-derived one-cell embryos (standard MEA).

**Results:**

BEA detected differences consistent with reduced developmental competence and embryo quality across 4118 bovine oocytes (≥ 4 independent cycles; ≥ 50 oocytes/group). Sperm selection media performed similarly. Regarding fertilization media, the bovine control yielded higher day 8 blastocyst rates than Vitrolife (*P* < 0.005), while Genea Biomedx exhibited superior blastocyst quality indicators than Vitrolife (*P* < 0.05). For embryo culture media, the bovine control outperformed Genea Biomedx, with Vitrolife performing intermediately (*P* < 0.05). Multiple kinetic, hatching, first lineage allocation and survival endpoints were reduced for Genea Biomedx (*P* < 0.05). Standard MEA found no significant differences, whereas IVF-MEA (1028 oocytes) detected a difference in day 4 blastocyst yield only.

**Conclusion:**

BEA revealed functional and quality differences between media not detected by mouse assays, supporting BEA to strengthen safety assessment while reducing animal sacrifice.

## Introduction

Human-assisted reproduction relies on culture media and a range of embryo-contact devices, the safety of which is usually confirmed through pre-market and lot-release quality control [1, 2]. For decades, the standard mouse embryo assay (MEA) has been the primary biological test used to screen-assisted reproduction devices for embryotoxicity [3–5]. However, routine mouse embryo testing is widely criticized for its limited discriminatory power under current acceptance thresholds [6], as it often classifies tested lots as non-toxic while failing to reveal sublethal effects that could impair embryo competence. Under the guidance commonly applied by the FDA [7], lots are typically accepted when at least 80% of the embryos reach the expanded blastocyst stage. This criterion is often met and may mask meaningful differences between lots or products. This concern is exacerbated by the lack of standardization of key parameters (culture duration, embryo numbers, and endpoints) across laboratories, as well as by the known physiological differences between mouse and human early development [8–10].

In parallel, societal and regulatory pressure to reduce animal use in testing has intensified the need for approaches that are more informative and can be implemented in quality control settings [11, 12]. A practical alternative should preserve the conceptual strengths of embryo-based testing (i.e., integrating complex cell-microenvironment interactions) while improving sensitivity and reducing reliance on animals bred and sacrificed for this purpose [13, 14]. In this context, validating a bovine embryo assay (BEA), which uses in vitro matured oocytes as its starting material rather than one-cell in vivo produced embryos as in the MEA, is a sensible option.

Indeed, bovine in vitro embryo production provides an abundant supply of material sourced from slaughterhouses, enabling larger sample sizes and a broader range of developmental and quality endpoints than are typically used in MEAs [13]. Here, we conducted a large-scale comparison of a BEA with the MEA currently used for testing assisted reproduction devices. Using identical batches of sperm, fertilization, and embryo culture media from two commercial suppliers, we quantified the success of development, the kinetics of development, the first lineage specification in the blastocyst, and the cryotolerance of bovine embryos. We also compared the ability of MEA to detect differences across the same test articles.

## Materials and methods

All chemicals were purchased from Sigma-Aldrich Quimica, S.A. (Madrid, Spain) unless otherwise indicated.

### Study overview

**Fig. 1:**
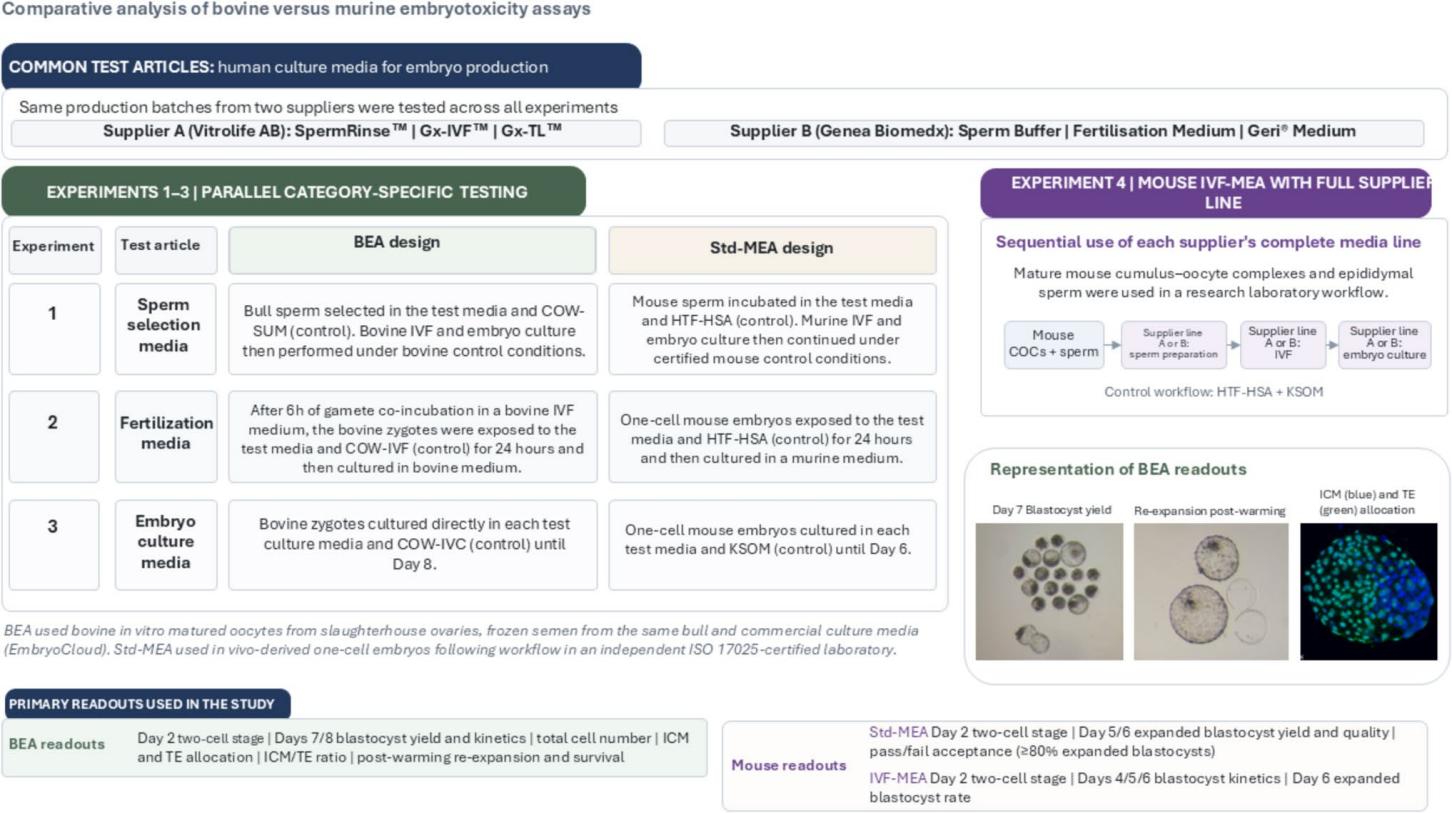
Experimental design used to compare bovine embryo assay (BEA) and murine embryo assay (MEA) for embryotoxicity-based assays. The same production batches of sperm selection, fertilization, and embryo culture media from two suppliers (Vitrolife and Genea Biomedx) were tested. Experiments 1–3 compared BEA and standard MEA (Std-MEA) in parallel for each media category. Experiment 4 evaluated the full supplier workflow in MEA including fertilization process (IVF-MEA). HTF-HSA, human tubal fluid medium supplemented with human serum albumin; ICM, inner cell mass; TE, trophectoderm

Four experiments (Fig. 1) were performed to compare bovine and mouse embryo-based embryotoxicity testing across three categories of assisted reproduction culture media: sperm selection media, fertilization media, and embryo culture media. Experiments 1–3 evaluated each category separately using a BEA in parallel with the standard MEA (Std-MEA) performed by an independent certified laboratory. Experiment 4 evaluated the complete, sequential use of each supplier’s media line in a mouse assay that included in vitro fertilization (IVF-MEA).

### Test articles

The same production batches of culture media from two commercial suppliers were used throughout. One supplier was Vitrolife AB (Sweden) and the other supplier was Genea Biomedx (Sydney, Australia). Each supplier contributed one medium for sperm selection (SpermRinse™ and sperm buffer medium, respectively), one for fertilization (Gx-IVF™ and fertilization medium) and one for embryo culture (Gx-TL™ and Geri® Medium). Species-appropriate control media were used for bovine procedures (EmbryoCloud, Murcia, Spain) and for mouse procedures, as described below. The same batches of Vitrolife and Genea Biomedx culture media used for the experiments in the BEA were used for Std- and IVF-MEA.

### Bovine embryo assay (BEA)

Bovine ovaries from postpubertal crossbred beef heifers were obtained from a commercial abattoir and transported to the laboratory in warmed saline (38.5°C) within 2 h.

Cumulus-oocyte complexes (COCs) were aspirated from 2–8 mm antral follicles and selected based on morphology. The COCs were then rinsed in COW-WASH medium (EmbryoCloud, Murcia, Spain) before being matured in vitro in COW-IVM medium (EmbryoCloud) in groups of 50–55 in 500 µl for 22–24 h at 38.5 °C in a humidified atmosphere containing 5% CO_2_. In vitro fertilization (IVF) was performed using frozen-thawed semen from a single bull of known fertility after sperm selection by swim-up in COW-SUM medium (EmbryoCloud). In brief, one straw was thawed (38 °C, 30 s), and 165 µL semen was transferred to the bottom of a conical tube containing 1 mL pre-warmed COW-SUM medium. The tube was kept at a 45° angle for 1 h at 38.5 °C. After this time, 750 µL of the supernatant from the top was aspirated and centrifuged (300 *g*, 7 min), and 650 µL of the supernatant was then discarded. Sperm motility was checked in the remaining pellet, and only over 75% were used to inseminate COCs at a final concentration of 10^6^ cells/mL. Gametes were co-incubated in COW-IVF medium (EmbryoCloud) for 24 h at 38.5 °C under 5% CO_2_. As fertilization of bovine oocytes using human IVF media was unsuccessful, to test IVF media, gametes were co-cultured for 6 h in COW-IVF medium (EmbryoCloud) and putative zygotes transferred for a further 24 h to IVF media from both suppliers (Vitrolife and Genea Biomedx) and control (EmbryoCloud).

After coculture time, putative zygotes were denuded by vortex (4 min, 20 Hz) in COW-WASH medium and cultured under oil in groups of 25 in 50 µl COW-IVC medium (EmbryoCloud) at 38.5 °C in a humidified atmosphere with 5% CO_2_ and 5% O_2_ for up to 8 days.

Endpoints for BEA included cleavage rate at day 2, cumulative blastocyst yield at days 7 and 8, and morphological developmental stage distribution at days 7 and 8 [15]. Blastocyst quality was assessed by differential cell staining [16] to quantify total cell number, inner cell mass cell number (ICM), trophectoderm cell number (TE), and the inner cell mass to trophectoderm ratio (ICM/TE). Briefly, embryos were permeabilized overnight at 4 °C (0.5% Triton-X and 0.05% Tween in DPBS), blocked overnight at 4 °C (10% goat serum and 0.05% Tween in DPBS), and incubated overnight at 4 °C in anti-CDX2 primary antibody (Biogenex AM392 RTU, San Ramon, USA) to mark the TE cells. Finally, embryos were incubated for 1 h at room temperature in 1:500 donkey anti-mouse Alexa Fluor® 488 (715–546-150, Jackson ImmunoResearch, Pennsylvania, USA), mounted in Vectashield (DAPI; Vector Laboratories, California, USA), and evaluated under fluorescence microscopy. All embryos were stained outside stages where compaction would make individual nuclei difficult to resolve, and ICM and TE cells in each blastocyst were counted independently three times by experienced personnel; the average value was then used for analysis.

Cryotolerance was evaluated by vitrifying day 7 expanded blastocysts (quality grades 1–2) and recording re-expansion of the blastocoel and survival at 3 h and 24 h after warming [17]. Briefly, embryos were vitrified in a cryotop device (Kitazato, Fujinomiya, Japan) as previously described [18, 19] using COW-WASH supplemented with 20% fetal bovine serum as holding medium and performing all the steps at 38.5 °C. After warming, blastocysts were washed in pre-equilibrated COW-IVC medium and cultured at 38.5 °C, humidified atmosphere with 5.0% CO_2_ and 5.0% O_2_.

### Mouse embryo assays (MEA)

Standard mouse embryo assay (Std-MEA): The Std-MEA was conducted by Embryotools S.L. (Barcelona, Spain), an ISO 17025-certified independent laboratory, following current regulatory guidance [7, 20].

To test the embryotoxicity of sperm selection medium, fresh mouse epididymal sperm were obtained from fertility-proven F1 hybrid males (B6/CBA) in commercial human tubal fluid (EmbryoMax® human tubal fluid) supplemented with 0.4% human serum albumin (HTF-HSA). The sperm were diluted 1:20 with prewarmed and pre-equilibrated SpermRinse™ (Vitrolife) and prewarmed sperm buffer (Genea Biomedx), disposed in 50-µL drops covered by oil and incubated for 2 h at 37.3 °C under 5% CO_2_. Then freshly COCs collected from females with the same genetic background were released in the drops with sperm and after 4 h of gamete coincubation; fertilized oocytes were selected and cultured in KSOM medium (EmbryoMax® Advanced KSOM Embryo Medium) up to Day 5 at 37.3 °C, 6% CO_2_ and 7% O_2_ conditions.

One-cell mouse embryos freshly collected from F1 hybrid females B6/CBA crossed with males from the same genetic background were cultured in IVF and IVC test media for durations aligned with each medium’s intended use. For IVF media, embryos were cultured for 24 h in IVF media from both suppliers and HTF-HSA (control) and then continued culture in KSOM medium up to Day 6. For IVC media, one-cell embryos were cultured in test media and KSOM medium (control) up to Day 6. Acceptance criteria and scoring followed the certified laboratory workflow, including a requirement that at least 80% of embryos develop to the expanded blastocyst stage within the specified culture window. Cleavage and blastocyst development and morphology were assessed using the laboratory’s certified workflow and acceptance criteria.

Mouse assay including IVF (IVF-MEA): A research laboratory (INIA-CSIC, Madrid, Spain) conducted a mouse assay involving sperm preparation, IVF of freshly collected in vivo-matured mouse COCs (B6CBAF1), and embryo culture using each media line sequentially.

Epididymal sperm were collected from B6CBAF1 (C57BL/6xCBA) males (8–20 months) in M2 manipulation medium supplemented with 0.1% bovine serum albumin (BSA). One of the epididymis from two different males were combined to be washed-out in each test media to avoid male effect in the IVF and two epididymis from a third male were washed-out in HTF-HSA (control). Sperm were selected by swim-up for 30 min at 37 °C and 5% CO_2_ in an oil-covered drop of 500 µL of each test media. Metaphase II oocytes were collected from the oviducts of superovulated B6CBAF1 females (6–8 weeks). For IVF, COCs were released from ampulla in each specific test medium for IVF and HTF-HSA (control) and placed into a fertilization drop. Co-incubation of gametes was done at 1 × 10^6^ sperm/mL with 30–50 COCs in a 500-µL drop of each test media covered by oil for 5 h at 37 °C, 5% CO_2_, and humidity. After incubation, zygotes were moved to 30-µL drops of each test medium and KSOM (control) until day 6.

### Ethics and oversight

Institutional Review Board status: not applicable; no human participants, clinical specimens, or identifiable patient data were used.

Bovine material consisted of slaughterhouse by-products obtained from routine commercial operations; no live animals were handled or subjected to experimental procedures for this study.

Institutional Animal Care and Use Committee approval: all procedures for IVF-MEA were performed in accordance with European and national legislation for animal research and were approved by the institutional ethics committee and competent authority under protocol PROEX 137.2/21.

### Statistical analysis

Data are expressed as the mean ± standard error of the mean (SEM). Rates for cleavage at D2, blastocyst formation at D7 and D8, and survival and re-expansion after at 3 and 24 h after warming were assessed together with TCN, ICM, TE, and ICM/TE per blastocyst. As the official reports for the Std-MEA did not include the number of cells in each blastocyst, but rather the mean number, it was not possible to compare the experimental groups for this variable, and a *P*-value could not be calculated. All percentages were modeled according to the binomial model of variables and arcsin transformation to achieve normal distribution. One-way ANOVA was used considering the culture media (sperm selection, IVF, and embryo culture) as the main factor. When ANOVA revealed a significant effect, values were compared by the least significant difference pair wise multiple comparison post hoc test (Tukey). Differences were considered statistically significant at *P* < 0.05. The analysis was conducted using SPSS v28 (SPSS Inc., Chicago, IL, USA).

## Results

### Bovine dataset and replicate structure

Across experiments 1–3, a total of 4118 bovine oocytes were used, with at least four independent IVF cycles per medium category and supplier and at least 50 oocytes per group per replicate. The distribution by experiment was 1249 oocytes for sperm selection media, 1392 oocytes for fertilization media, and 1477 oocytes for embryo culture media. MEAs were performed on the same batches of media in parallel (51 oocytes in sperm selection testing, 75 one-cell embryos for fertilization media testing, and 80 one-cell embryos for embryo culture media testing in Std-MEA). As for IVF-MEA, a total of 1028 oocytes were used to test each supplier’s media line sequentially.

### Experiment 1: Sperm selection media

**Fig. 2:**
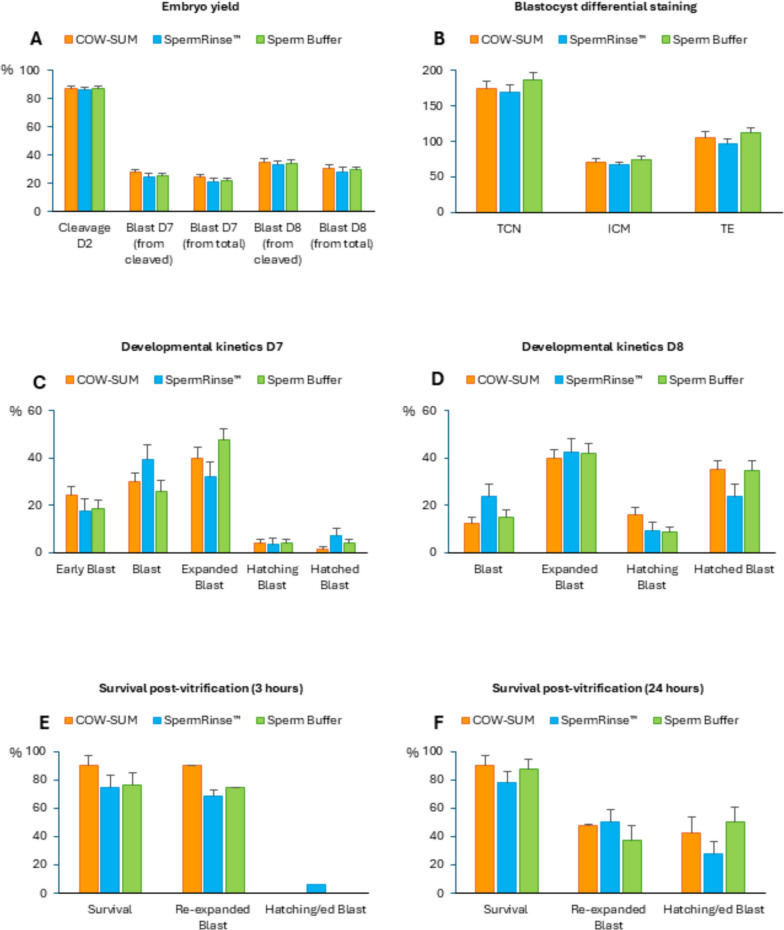
Embryotoxicity and functionality through the BEA of two human sperm selection media. SpermRinse™ (Vitrolife) and sperm buffer (Genea Biomedx) media are compared with a bovine sperm selection medium (control group, COW-SUM, EmbryoCloud). **A** Embryo yield with cleavage rate at Day 2 and blastocysts rates at Days 7 and 8. **B** Total number of cells per blastocyst (TCN), number of cells in the inner cell mass (ICM), number of cells in the trophectoderm (TE) for bovine blastocysts on Day 7 (expanded category) or Day 8 of culture (blastocyst, expanded, hatching, and hatched categories). **C** Blastocysts developmental kinetics at Day 7. **D** Blastocysts developmental kinetics at Day 8. **E** Post-vitrification survival rate and blastocyst re-expansion at 3 h. **F** Post-vitrification survival rate and blastocyst re-expansion at 24 h. Blast, blastocyst. Data are expressed as the mean ± standard error of the mean (SEM)

When bull sperm were selected using sperm selection media from Vitrolife (SpermRinse™) or  Genea Biomedx (sperm buffer medium) and then used to fertilize bovine oocytes under identical bovine fertilization and embryo culture conditions, no differences were observed relative to the bovine control medium for cleavage, blastocyst yield at days 7 and 8 or developmental kinetics (Fig. 2A, C, and D).

Blastocyst quality metrics, including total cell number, inner cell mass cell number, trophectoderm cell number (Fig. 2B) and inner cell mass to trophectoderm ratio (0.7 ± 0.1, 0.7 ± 0.1, and 0.7 ± 0.1, respectively, for control, Vitrolife, and Genea Biomedx groups) were comparable across groups Cryotolerance, assessed by re-expansion and survival at 3 h and 24 h after warming, also did not differ among groups (Fig. 2E, F).

The Std-MEA results for the same sperm selection media batches likewise showed no differences relative to control (Table 1).

**Table 1:** Embryo yield a*n*d ki*n*etics obtai*n*ed in the standard mouse embryo assay (Std-MEA) with sperm selection media from two different suppliers (SpermRinse™ from Vitrolife and sperm buffer from Genea Biomedx)

	*n*	Day 2 Two-cell stage *n* (%)	Day 5 Expanded blastocyst* n (%)	Good quality (morphology) blastocysts *n* (%)	Result
Control	16	15 (93.7)	15 (93.7)	11 (73.3)	Passed
SpermRinse™	15	15 (100)	15 (100)	8 (53.3)	Passed
Sperm Buffer	20	20 (100)	18 (90)	15 (83.3)	Passed
P-value		0.342	0.476	0.411	

*Blastocyst rates calculated from cleaved zygotes. Different letters denote significant differences between groups (*P* < 0.05). NS, not significant. HTF-BSA and KSOM media were used in control group. Data are expressed as the mean ± standard error of the mean (SEM)

### Experiment 2: Fertilization media

Human fertilization media did not support sperm penetration into bovine oocytes under the conditions tested; therefore, embryotoxicity was assessed in a manner analogous to Std-MEA by exposing bovine zygotes to test fertilization media for 24 h and then continuing culture in bovine embryo culture medium.

**Fig. 3:**
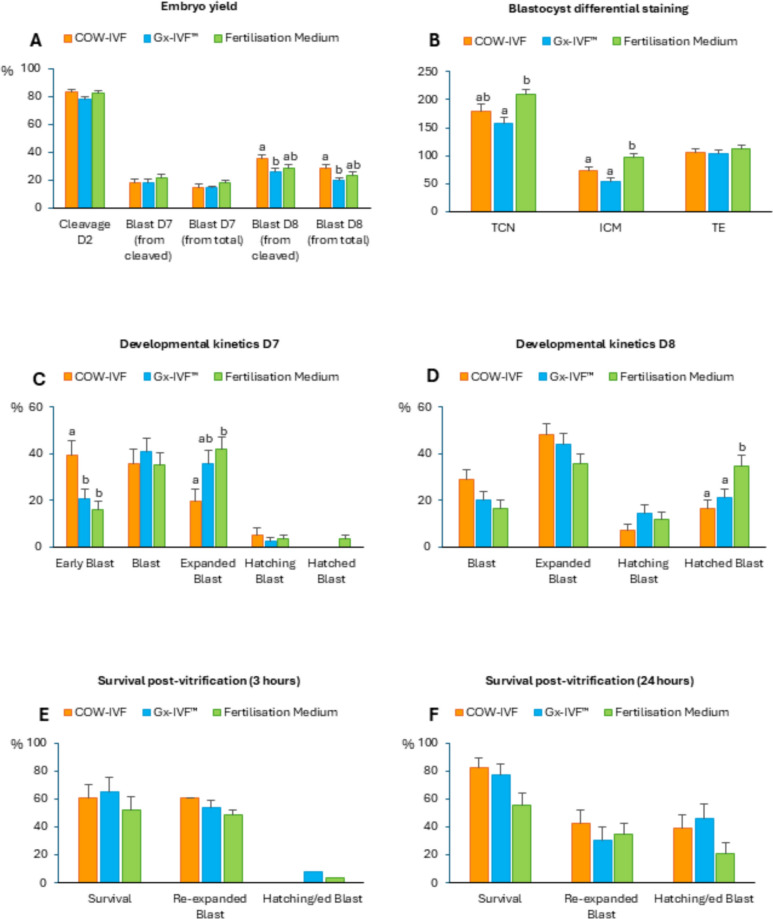
Embryotoxicity and functionality through the BEA of two human IVF media. Gx-IVF™ (Vitrolife) and Fertilization medium (Genea Biomedx) media are compared with a bovine sperm selection medium (control group, COW-IVF, EmbryoCloud). **A** Embryo yield with cleavage rate at Day 2 and blastocysts rates at Days 7 and 8. **B** Total number of cells per blastocyst (TCN), number of cells in the inner cell mass (ICM), number of cells in the trophectoderm (TE) for bovine blastocysts on Day 7 (expanded category) or Day 8 of culture (blastocyst, expanded, hatching, and hatched categories). **C** Blastocysts developmental kinetics at Day 7. **D** Blastocysts developmental kinetics at Day 8. **E** Post-vitrification survival rate and blastocyst re-expansion at 3 h. **F** Post-vitrification survival rate and blastocyst re-expansion at 24 h. Blast, blastocyst. Data are expressed as the mean ± standard error of the mean (SEM)

Under this design, the day 8 blastocyst yield was lower in Vitrolife’s fertilization medium (Vitrolife, Gx-IVF™), compared with the bovine control (*P* < 0.005), while the Genea Biomedx (Genea; sperm buffer medium) showed intermediate yield (Fig. 3A).

Beyond yield, the bovine embryo assay identified differences in embryo quality: total cell number, inner cell mass cell number (Fig. 3B), and the inner cell mass to trophectoderm ratio (0.8 ± 0.1, 0.6 ± 0.1, and 0.9 ± 0.1, respectively for control, Vitrolife, and Genea Biomedx groups) were lower after exposure to Vitrolife fertilization medium compared with Genea Biomedx (*P* < 0.05).

Developmental stage distribution also differed, with a higher proportion of hatched blastocysts in the better-performing fertilization medium (Genea Biomedx) at day 8 (*P* < 0.005) (Fig. 2D). Post-warming survival did not differ significantly among groups (Fig. 3E, F).

In contrast, Std-MEA did not detect significant differences between fertilization media and control under its routine readouts, and both media met acceptance criteria (Table 2).

**Table 2:** Embryo yield a*n*d ki*n*etics obtai*n*ed in the standard mouse embryo assay (std-MEA) with IVF media from two different suppliers (Gx IVF™ from Vitrolife and fertilization Medium from Genea Biomedx)

	*n*	Day 2 Two-cell stage *n*(%)	Day 5 Expanded blastocyst* n (%)	Good quality (morphology) blastocysts *n*(%)	Result
Control	15	15 (100)	15 (100)	14 (93.3)	Passed
Gx IVF™	30	30 (100)	30 (100)	26 (86.7)	Passed
Fertilization Medium	30	30 (100)	30 (100)	24 (80.0)	Passed
*P*-value		-	-	0.485	

*Blastocyst rates calculated from cleaved zygotes. Different letters denote significant differences between groups (*P* < 0.05). NS, not significant. HTF-BSA and KSOM media were used in control group. Data are expressed as the mean ± standard error of the mean (SEM)

### Experiment 3: Embryo culture media

**Fig. 4:**
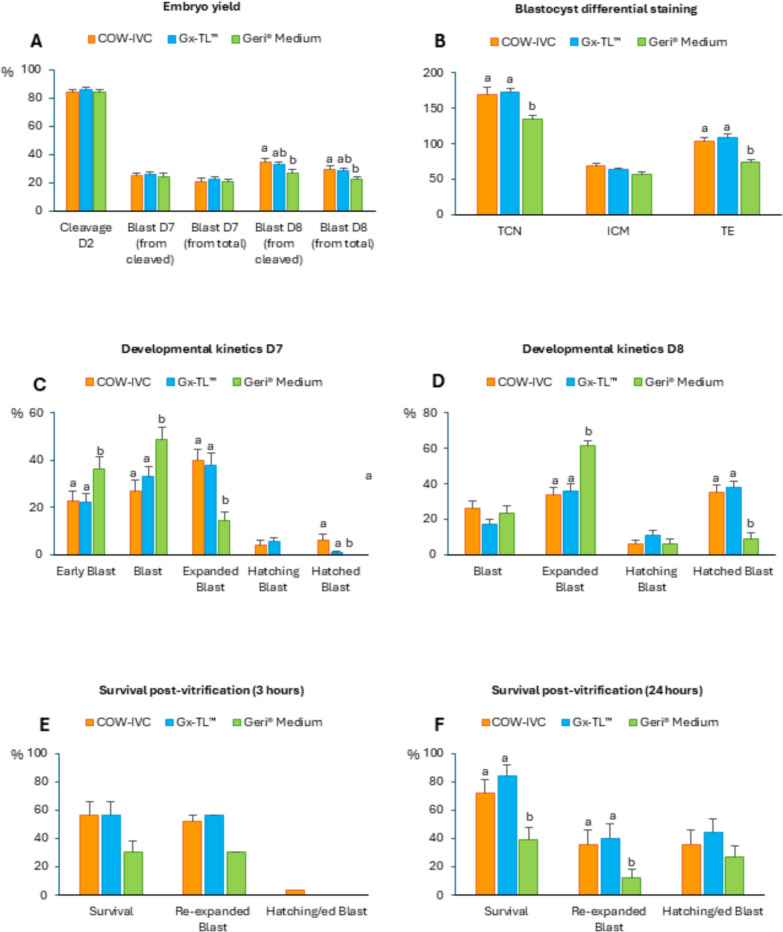
Embryotoxicity and functionality through the BEA of two human embryo culture media. Gx-TL™ (Vitrolife) and Geri® Medium (Genea Biomedx) media are compared with a bovine sperm selection medium (control group, COW-IVC, EmbryoCloud). **A** Embryo yield with cleavage rate at Day 2 and blastocysts rates at Days 7 and 8. **B** Total number of cells per blastocyst (TCN), number of cells in the inner cell mass (ICM), number of cells in the trophectoderm (TE) for bovine blastocysts on Day 7 (expanded category) or Day 8 of culture (blastocyst, expanded, hatching, and hatched categories). **C** Blastocysts developmental kinetics at Day 7. **D** Blastocysts developmental kinetics at Day 8. **E** Post-vitrification survival rate and blastocyst re-expansion at 3 h. **F** Post-vitrification survival rate and blastocyst re-expansion at 24 h. Blast, blastocyst. Data are expressed as the mean ± standard error of the mean (SEM)

When bovine zygotes were cultured for up to 8 days in embryo culture media from the two suppliers, the bovine embryo assay detected differences consistent with impaired developmental competence and reduced embryo robustness in Genea Biomedx’s medium (Genea Biomedx; Geri® Medium). Specifically, day 8 blastocyst yield was lower in Genea Biomedx compared with the bovine control (*P* < 0.05), and Vitrolife (Vitrolife, Gx-TL™) showed intermediate performance (Fig. 4A).

As for blastocyst quality endpoints, total cell number, trophectoderm cell number (Fig. 4B), and the inner cell mass to trophectoderm ratio (0.75 ± 0.04, 0.67 ± 0.03, and 0.86 ± 0.07 respectively for control, Vitrolife and Genea Biomedx) were reduced in Genea Biomedx medium (*P* < 0.05). Developmental kinetics were slower in the Genea Biomedx medium, with fewer hatched blastocysts at days 7 and 8 (*P* < 0.05) (Fig. 4C, D). These sublethal effects translated into reduced cryotolerance, with survival at 24 h after warming approximately 40% in the Genea Biomedx medium versus more than 70% in the Vitrolife medium and the bovine control (*P* < 0.05) (Fig. 4E, F).

As in Experiment 2, Std-MEA did not detect differences between embryo culture media and control, and both media met acceptance criteria (Table 3). Total cell number per blastocyst was similar between groups (206.9 ± 19.2, 174.8 ± 22.9, and 180.2 ± 41.2 respectively for Control, Vitrolife, and Genea Biomedx).

**Table 3:** Embryo yield and kinetics obtained in the standard mouse embryo assay (std-MEA) with embryo culture media from two different suppliers (GX-TL**™** from Vitrolife and Geri **®**Medium from Genea Biomedx)

	*n*	Day 2 Two-cell stage *n* (%)	Day 5 Expanded blastocyst* n (%)	Good quality (morphology) Blastocysts *n*(%)	Result
Control	16	16 (100)	16 (100)	15 (93.7)	Passed
Gx-TL™	32	32 (100)	31 (96.9)	26 (83.9)	Passed
Geri® Medium	32	31 (96.88)	31 (96.9)	27 (87.1)	Passed
*P*-value		0.478	0.781	0.526	

*Blastocyst rates calculated from cleaved zygotes. Different letters denote significant differences between groups (*P* < 0.05). NS, not significant. HTF-BSA and KSOM media were used in control group. Data are expressed as the mean ± standard error of the mean (SEM)

### Experiment 4: mouse embryo assay including in vitro fertilization (IVF-MEA)

In a mouse assay that included sperm preparation, fertilization, and embryo culture performed sequentially using each supplier’s media line (1028 oocytes across four replicates), overall developmental rates to blastocyst were similar between suppliers (Table 4). A small shift toward earlier blastocyst formation was observed for Genea Biomedx, but the assay did not reproduce the separation between embryo culture media that was evident in the bovine embryo assay.

**Table 4:** Embryo yield and kinetics obtained in the IVF mouse embryo assay (IVF-MEA) performed with the sequential use of two supplier’s (Vitrolife and Genea Biomedx) media lines

		Embryo yield		Embryo kinetics		
Group	*n*	Day 2 Two-cell stage *n* (%)	Day 6 Expanded blastocyst stage** n (%)	Day 4 Blastocyst stage *n*(%)	Day 5 Blastocyst stage *n* (%)	Day 6 Blastocyst stage *n* (%)
Control*	190	91 (47.9 ± 3.6)	32 (35.2 ± 5.0) a	4 (4.1 ± 2.2) a	27 (26.9 ± 4.8)	1 (1.1 ± 1.1)
Vitrolife	389	179 (46.0 ± 2.5)	84 (46.9 ± 3.7) ab	41 (24.6 ± 3.2) c	31 (20.1 ± 3.0)	2 (1.7 ± 0.9)
Genea Biomedx	449	212 (47.2 ± 2.3)	122 (57.5 ± 3.4) b	75 (38.2 ± 3.3) b	31 (18.0 ± 2.7)	2 (0.9 ± 0.7)
*P*-value		NS	0.001	< 0.001	NS	NS

*Control embryos were produced by using HTF-BSA and KSOM media

**Blastocyst rates calculated from cleaved zygotes. Different letters denote significant differences between groups (*P* < 0.05). NS, not significant. Data are expressed as the mean ± standard error of the mean (SEM)

## Discussion

This comparative analysis shows that, under routine designs and acceptance thresholds, MEAs may have limited sensitivity to discriminate between assisted reproduction culture media that differ in their effects on embryo competence. Across fertilization and embryo culture media, neither the Std-MEA nor the MEA including IVF identified relevant differences between suppliers despite bovine embryo assay findings that were consistent, statistically supported, and biologically meaningful, including altered developmental kinetics, different first lineage allocation, and diminished cryotolerance.

Several features of current mouse embryo testing likely contribute to low discriminatory power. The standard assay typically relies on a small number of embryos and a restricted set of endpoints dominated by morphology-based blastocyst scoring [7, 8]. In addition, the use of in vivo-derived embryos bypasses potential sensitivity at earlier stages, including sperm exposure and fertilization, and short exposure windows can further reduce the likelihood of detecting sublethal toxicity [9]. Finally, the std-MEA relies on the use of specific mouse strains that are crossed to maintain a specific genetic background, despite different studies which propose that only a pooled sample of different mouse strains can be used for comprehensive media MEA testing and that embryos from outbred mice may be more sensitive [21, 22].

These limitations are compounded by acceptance criteria that can be met by most lots [7], which supports lot release but does not necessarily maximize the ability to detect performance differences among devices [14].

By contrast, the bovine embryo assay leverages a large number of oocytes that are readily available as slaughterhouse by-products, enabling higher statistical power without breeding and sacrificing animals for testing [13, 14] [23].

A further advantage of the BEA for assay standardization is that sperm can be obtained from the same bull and used in quality-controlled batches, whereas in the MEA, sperm are typically collected from a different male mouse in each experiment, adding an additional source of biological variability. Moreover, the present study used defined production batches of each medium in order to minimize lot-to-lot variability as a confounding factor when comparing assay performance. Therefore, the study was not designed to evaluate batch-to-batch consistency, shelf-life, storage duration, or controlled degradation within the same medium. These aspects should be addressed in future validation studies, including multiple lots, stability-related designs, and inter-laboratory ring trials.

Crucially, the large sample size allows the bovine workflow to include endpoints related to embryo robustness, such as first lineage allocation and post-warming survival, without raising ethical issues. These endpoints are not captured by routine mouse embryo testing. Although incorporating them into MEA is plausible, it would incur the additional cost of purpose-bred laboratory animals. The relatively large number of bovine oocytes used here reflects the exploratory and comparative nature of this first direct evaluation of BEA, Std-MEA, and IVF-MEA across several media categories and endpoints. Once the most informative endpoints and expected effect sizes are established, future work should define reduced sample sizes by formal power calculations and streamline the assay for routine quality-control use.

Finally, the bovine assay is not restricted to a single inbred strain with homogeneous genetics, and bovine in vitro embryo production outcomes are consistently reported within ~ 30–40% expanded blastocysts on Day 8 across laboratories and countries [24–27].

Sperm selection media performed similarly for Vitrolife and Genea Biomedx. However, differences were observed in the IVF and embryo culture media from the two suppliers. Regarding the IVF media, identifying plausible explanations for the observed variations in BEA performance in this study requires consideration of both qualitative and quantitative composition. Analytical profiling studies have shown that these commercially available IVF media differ markedly in their concentrations of energy substrates, amino acids, and inorganic ions [28–30], and that such compositional differences can measurably influence blastocyst development in mouse models [29, 31]. In the present study, it is hard to pinpoint which specific component(s) in Vitrolife versus Genea Biomedx media drive any BEA-related differences in blastocyst quality because publicly available formulation details are limited. Genea Biomedx media trace back to Mortimer’s STF/Sydney IVF lineage [32], whereas Vitrolife media stem from Gardner and Lane’s sequential “back-to-nature” approach [33]. Both appear to be low-glucose formulations, but the most consistent compositional contrast is the lactate:pyruvate (L:P) ratio: Vitrolife is markedly lactate-rich (high L:P), while Genea Biomedx is relatively more pyruvate-rich with a more balanced L:P[29, 30]. Also, Genea Biomedx reports 5 mg/mL HSA, while Vitrolife has been indicated at ~ 10 mg/mL in earlier manuals [34]. Finally, Vitrolife includes a triple antioxidant system (acetyl-L-carnitine, alpha-lipoic acid, N-acetyl-L-cysteine), which could mitigate redox effects associated with high lactate. Therefore, L:P ratio, HSA, and antioxidants (among other factors) are plausible drivers of the differences found, but targeted experiments are needed to confirm causality.

For embryo culture (IVC) media, outcomes clearly favored Vitrolife under a continuous, no-renewal protocol (up to 8 days): Its IVC medium yielded more blastocysts, faster Day 7–8 kinetics, > 3 × more hatched blastocysts, higher cell numbers (especially TE), a higher ICM/TE ratio, and > 2 × better survival/re-expansion after vitrification than Genea Biomedx. As with IVF media, published comparisons suggest two distinct formulation strategies: Vitrolife-type systems are typically lactate-dominant, whereas Genea Biomedx–derived media show lower lactate and relatively higher pyruvate [29]. Marked differences are also reported in amino acids (sequential essential AA introduction in Vitrolife vs earlier inclusion and much higher glycine/taurine in Genea Biomedx, largely linked to HSA) and smaller but consistent differences in salts (Na/K/Mg slightly higher in Vitrolife-type media)[30]. Again, these compositional contrasts may or may not underlie the BEA performance differences, so targeted component-by-component studies are needed.

The last experiment, where IVF-MEA was performed with large numbers of mouse gametes (> 1000) in several replicates, only detected minor differences. A limitation of the IVF-MEA experiment is the absence of a direct comparison with the bovine embryo assay due to the fact that IVF-MEA used all three media together, whereas the bovine assay involved testing them separately. IVF-MEA exhibited a higher proportion of Day-4 blastocysts with the Genea Biomedx media, whereas the bovine test indicated that Genea Biomedx IVF medium primarily enhanced blastocyst quality in terms of total cell number and the proportion of hatched blastocysts. The potential for these overlapping advantages to persist during culture in IVF-MEA may have obscured the superior performance of the Vitrolife embryo culture medium, as observed in the bovine test.

Beyond the analytical differences observed between assays, the bovine model also presents biological features that are closer to human preimplantation development than the murine model, including a longer developmental timeline to the blastocyst stage, a monovulatory reproductive strategy, and greater similarities in embryonic genome activation timing and metabolic regulation. In addition, bovine ovaries can be obtained as a by-product of the meat industry, avoiding the need to breed and sacrifice animals specifically for the assay.

Collectively, these characteristics position the BEA as a pragmatic candidate for enhancing the sensitivity of embryotoxicity assessment, thereby aligning with the principles of replacement, reduction, and refinement in animal research [35].

This study also highlights considerations for implementation. Human fertilization media were not suitable for bovine fertilization in our hands, requiring an exposure design focused on zygote culture, analogous to current mouse embryo testing. We evaluated two suppliers and specific production batches; broader testing, including ring trials across laboratories, will be required to define robust performance benchmarks, acceptance criteria and reference materials. Finally, the present work was not designed to link assay outcomes with clinical outcomes; future studies could explore how sensitive embryo quality endpoints relate to clinically relevant performance measures.

Overall, these data support the use of a BEA as a more sensitive embryo-based approach to detect embryotoxicity and functional impairment in assisted reproduction culture media, and potentially other embryo-contact devices.

## Conclusions

The standard MEA, as routinely implemented, showed limited ability to discriminate between assisted reproduction culture media batches that differed in their effects on embryo development and robustness. A BEA using slaughterhouse-derived oocytes detected differences in developmental kinetics, first lineage allocation, and cryotolerance that were not revealed by mouse assays. These findings support the bovine model as a practical, more sensitive option to redefine embryotoxicity safety standards for assisted reproduction devices, pending broader inter-laboratory validation and consensus acceptance criteria.

## Data Availability

All summary data supporting the findings of this study are included in this published article. The underlying raw datasets generated and/or analysed during the current study are not publicly available but are available from the corresponding author on reasonable request.
